# Possible implications of sea level changes for species migration through the Suez Canal

**DOI:** 10.1038/s41598-020-78313-2

**Published:** 2020-12-03

**Authors:** Eli Biton

**Affiliations:** Department of Physical Oceanography, Israel Oceanographic and Limnological Research, 31080 Haifa, Israel

**Keywords:** Animal migration, Physical oceanography, Climate-change impacts

## Abstract

The Mediterranean and Red Sea, which were connected via the Suez Canal during the 19th century after eons of separation, host two distinctive ecosystems. Species invasion through the Suez Canal from the Red Sea vastly influences the ecology of the Mediterranean, but the level of reverse migration is assumed to be negligible. We present the first reconstructed flow transport record through the canal during the period 1923–2016. According to this reconstruction, the flow intensity and direction through the canal are strongly influenced by seasonal and long-term sea-level changes, which could also play a role in the characteristics of species migration through it. Our record not only supports previous observations of the unidirectional invasion until the 1980s and the accelerated species migration rates to the Mediterranean ever since, but also suggest that southward migration could have become possible since the early 1980s. The southward flow was primarily enhanced by Indian Ocean cooling and the Eastern Mediterranean Transition in deep water formation during the period 1980–2000. It was then gradually reduced by accelerated sea-level rise in the northern Indian Ocean.

## Introduction

**Figure 1:**
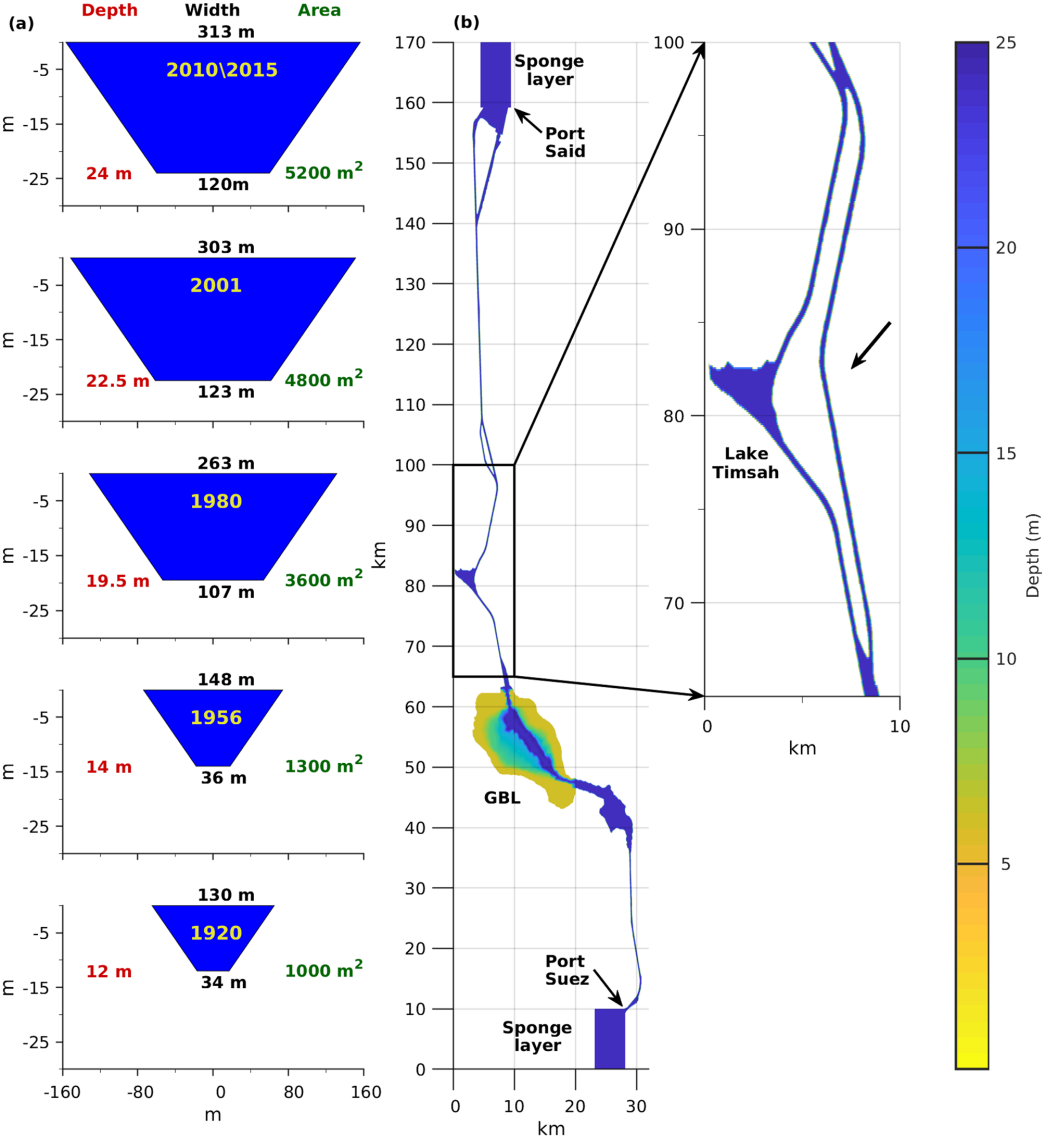
Dimensions of the Suez Canal (SC) used to simulate its different development stages. (**a**) Increased cross-sectional dimensions of the SC proper (apart from the lakes region) over time. Years are shown in yellow. Red, black, and green values indicate the depth, width at the bottom and at the surface, and the area of each cross-section, respectively. (**b**) Bathymetry of the SC used to simulate the conditions for the year 2010. The inset shows the 35-km section that was added to the SC (indicated by the black arrow) to simulate the latest major construction stage completed in 2015.

The Suez Canal (SC) spans over 165 km of sandy desert between Port Said on the Mediterranean Sea (MS) and Port Suez on the Gulf of Suez, northern Red Sea (RS) (Fig. 1). Since its opening, the canal was expanded and deepened a number of times to overcome the increasing volume of commercial shipping and the increasing size and draughts of modern ships (Fig. 1a). The SC is a dynamically complex body of water, connecting several lakes with widely diverse salinities to two marginal seas with very different thermohaline properties and sea level conditions. It is also a well-known conduit for marine species invasions between the RS and the MS. The recorded migration is highly asymmetric, with $\sim$ 500 species known to have entered the MS through the SC (also known as non-indigenous species), resulting in the continuous loss of important native populations in the MS, including habitat-forming ecosystem engineers^1^. However, only a few records exist of species successfully migrating in the opposite direction, from the MS to the RS. Most of the alien species that traverse the SC do so by drifting or swimming, where other pathways of introduction, such as transport via ballast water of ships, have less contribution^2–6^. The high salinity and the seasonal flow of the SC were assumed to pose an effective physical obstacle influencing the type of migrating species and their migration rates and direction (RS to MS or vice versa)^1,4,5,7–10^. However, the effects of SC dynamics and their response to its deepening and expansion over the years (Fig. 1a) on species migration between the two seas have not been studied rigorously. The asymmetric migration has been attributed to a dominant northward current through the SC from the RS to the MS, or to other factors such as environmental conditions and species adaptation^7^. Moreover, the accelerated rate of species invasion from the RS to the MS since the early 1980s has been related to either climate change (warming of the eastern Mediterranean) or a reduced physical barrier caused by the expansion of the SC over the years^11^. Over 150 years after its establishment^12^, the ecological effects of the SC are still increasing, resulting in challenges and concerns for the scientific community: for example, in 2015, a new 35-km bypass enabling two-way traffic (also knowns as “new Suez Canal”) was excavated in parallel to part of the original 165-km canal (Fig. 1b), which could result in a new wave of species invasion to the MS^13^.

In the current study, we combined 3D hydrodynamic models and sea-level (SL) observations to study the long-term dynamic variations in the SC and their potential impacts on species migration between the MS and the RS. Notably, in what follows we only consider species that traverse the Suez Canal by drifting or swimming, while alien species that crossed the Suez Canal by other modes of introduction, such as shipping, were probably negligibly affected by the SC dynamics and its physical conditions (currents, salinity, temperature, turbidity, etc). Previous works used simplified models to study the dynamics of the SC^14^ and/or its contribution to asymmetric migration^7^; however, these studies focused on short periods and lacked any climatic interpretations of their findings. Here, for the first time, we reconstruct a continuous record of the volumetric flow rate (hereafter simply indicated by transport) through the SC for the period 1923–2016. Our record accounts for the enlargement of the canal over the years, including the opening of the “new Suez Canal” in 2015, and the sea level difference between its two ends, thereby provides a strong physical basis and climatic context for the species migrations through the SC over this period. We show that in addition to general increase of flow transport due to the expansion of the SC over the years, the seasonal variability and long-term trends in flow transport through the SC are likely to be dominated by sea level (SL) variations in the RS and MS, which in turn potentially affect the species migrations between the two seas. Particularly, our reconstructed transport record through the SC suggests that the physical barrier to southward migration has reduced significantly since the 1980s, suggesting an enhanced migration to the RS ever-since.

## Flow transport record through the Suez Canal

**Figure 2:**
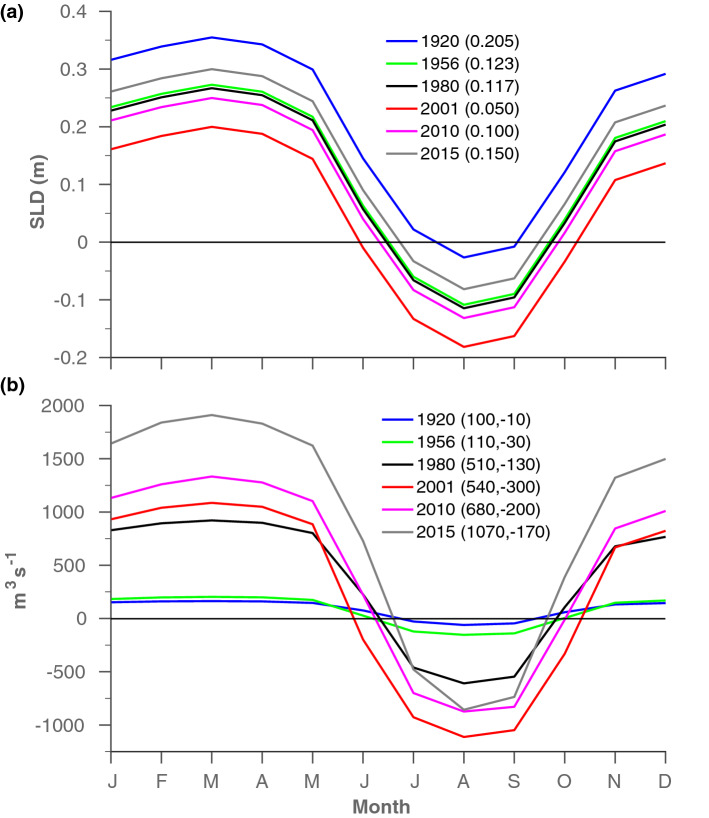
Seasonal sea level differences between Port Suez and Port Said (SLD) and modelled flow transport results during different construction stages of the Suez Canal (SC). Monthly mean values of (**a**) SLD and (**b**) modelled flow transport along the SC calculated from simulation R1920-R2015 (“Methods” section). Quantities in parentheses are the annual mean values of SLD in (**a**) and the flow transport to the north (positive values) and south (negative values) in (**b**). The SLD values in (**a**) were calculated based on SL seasonalities shown in Fig. 6a.

Only a few current measurements in the canal have been made publicly available, and thus most of the information regarding the SC dynamics was indirectly inferred from outdated hydrographic and sediment datasets^15^ and simplified models^7,14^. Our model results indicate that the along-canal flow switching from a northward flow during winter to a southward flow during the summer (Fig. 2b), where the seasonal variations of SL differences between the RS and MS sides of the SC (hereafter SLD, where SLD=SL$_{RS}$ − SL$_{MS}$) are the primary cause for the observed flow seasonality (“Methods” section and Fig. 7). In Port Said, the northern end of the SC (Fig. 1), the SL is affected by thermosteric effects attributed to seasonal surface heating and cooling and being high during summer and low in winter (Figs. 5 and 6a). In contrast, the SL at Port Suez and along the RS is suggested to be driven by the Indian Monsoon variability, showing maximal values during winter and minimal during summer (Figs. 4a, 6a and Supplementary Information). The above anti-correlated seasonal SL variabilities on both sides of the SC translate into positive SLD values during the winter that drive northward flow from the RS to the MS, and negative SLD values during summer that drive southward flow (Fig. 2). Similar seasonality existed during all expansion and deepening stages of the SC (Fig. 2b). Yet, in addition to the expected increasing seasonal amplitude of the flow transport as a result of the expansion of the SC (Fig. 2b), the relative significance of the two flow regimes, in terms of transport and seasonal duration, has changed over time, mirroring trends in the annual mean SLD that cause general shifts in the two SL seasonal signals (Fig. 2a and Fig. 6). For example, during high-stand annual mean SLD conditions, northward flow is more frequent (e.g., during the 1920s) but becomes comparable in magnitude and seasonal duration in both directions when the mean SLD decreases (e.g., during the 2000s). Overall, northward transport has increased, peaking in 2016 at $1070\,\text {m}^3\,\text {s}^{-1}$, which is an order of magnitude higher than a century ago (Fig. 2b). The first significant increase in northward flow occurred during the early 1980s after a major construction stage, with a second occurring in 2015 after the opening of the ‘new’ Suez Canal. In comparison, the summertime southward flow regime peaked in 2001 at 300 m$^3$s$^{-1}$, compared with only 10 m$^3$s$^{-1}$ during the 1920s. However, since 2001 the southern flow has been declining despite further construction in 2010 and 2015 (Fig. 2b). In addition, during the high-stand SLD conditions of the 1920s, the southward flow occurred from August to September. In comparison, during the low-stand SLD conditions in 2001 the duration of the southward flow extended and prevailed from June to October. Since 2001, the seasonal duration of southward flow shrunk and by 2015 occurred from July to September.

**Figure 3:**
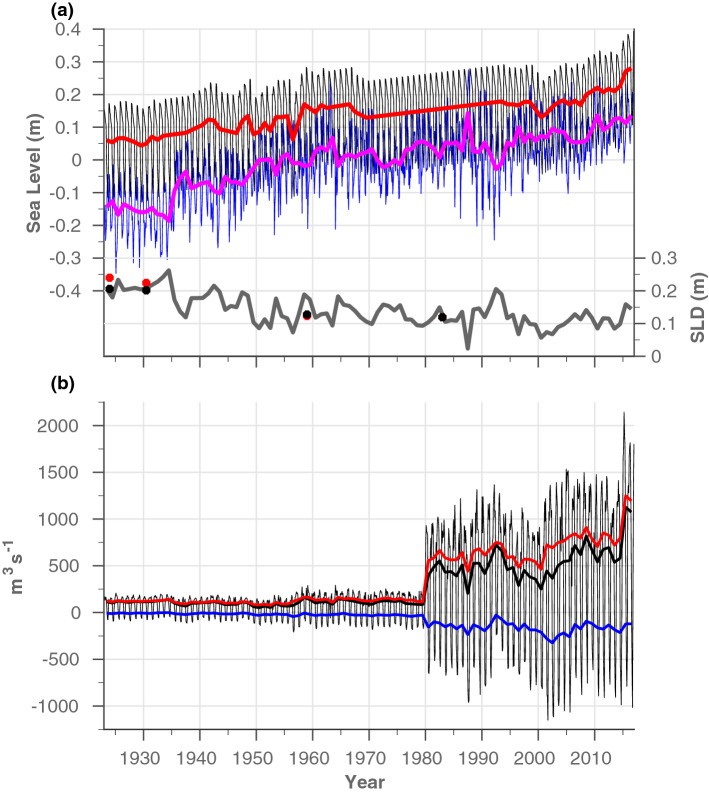
Reconstructed records of SL, SLD and flow transport through the SC. (**a**) left-axis: monthly (thin lines) and annual (thick lines) mean SL records at Port Suez (black and red, respectively) and at Port Said (blue and magenta, respectively); right-axis: annual mean SLD. Previous estimates of the mean SLD over the periods 1923–1925, 1924–1937, 1955–1963, and 1980–1986 are also indicated in red dots, compared with our calculations in black dots. (**b**) reconstructed monthly mean flow transport along the SC from 1923 to 2016 (thin black line) calculated from RMATRIX results. Thick lines represent the annual mean records of flow transport to the south (blue), north (red), and for the residual flow (black). See “Methods” section for more detail.

**Figure 4:**
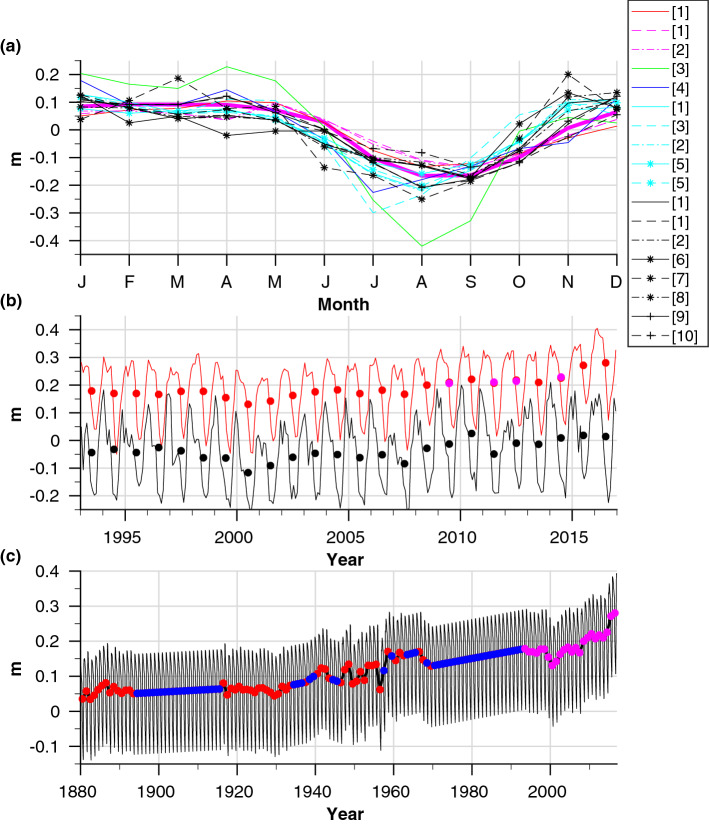
Processing of the SL record at Port Suez. (**a**) Monthly mean SL anomaly records taken from the GOA (red) and at five different places along the RS (Perim Island (magenta), Jeddah (green), Gizan (blue), Port Sudan (cyan), and Port Suez (black)) compared with the steric effect conditions at Perim Island (thick magenta line). Line styles represent different time periods/years of the measurements, and the numbers in the brackets refer to the following sources for the datasets: [1]^56^, [2]^71^, [3]^72^, [4]^55^, [5]^73^, [6]^52^, [7]^74^, [8]^57^, [9]^75^, and [10]^69^. (**b**) Monthly (solid lines) and annual (dots) means of the satellite ADT data from GOA (red) and northern RS (black). The annual mean SL values measured at the GOA using tidal gauges are also indicated (magenta dots). (**c**) Computed monthly (thin black line) and annual (thick black line) means SL records at Port Suez (also shown in Fig. 3a). The coloured dots illustrate the different sources contributing to the computational record, including in situ SL tidal gauge data from GOA (red)^53,54^, satellite ADT data from GOA (magenta), and the interpolated data (blue).

To further assess the possible variations in migration characteristics in the context of climate change, long-term SLD and flow transport records were reconstructed for 1923 to 2016 (Fig. 3, see “Methods” section for more details). Similar to the results presented in Fig. 2, the effects of SC widening and deepening over the years (Fig. 1) manifests as sharp increases in both southward and northward transport, especially during the early 1980s and 2015 (when the New Suez Canal was opened), yet clear trends in the seasonal flow are also evident during the intervals between the different construction stages (e.g., 1980–2001). These trends in the seasonal flow following long-term variations in the reconstructed SLD record, which are constrained by the applied long-term SL conditions in the RS and MS (Fig. 3). Each end of the SC is located at the far reaches of their respective marginal seas, therefore, the SL at either end is affected by regional and distant climatic processes. SL changes are not uniform around the globe, especially in semi-enclosed seas. The SL of the MS does not respond linearly to the open ocean, but rather exhibits decadal to inter-decadal variability associated with changes in its mass balance and steric contribution^16–20^. These variations are influenced by a variety of remote climatic modes, such as North Atlantic Oscillations (NAO)^19,20^ and regional processes, such as deep-water formation in the MS^21–23^. Little is known about SL variations and trends in the RS and their climate-related drivers. Satellite altimetry data indicate that SL trends in the northern RS and northern Indian Ocean (IO) are highly correlated (Fig. 4b and Supplementary Information). Moreover, our independent SLD calculations correspond to previous sporadic estimates (Fig. 3b and “Methods” section), indicating that the SL in the RS follows that of the northern IO, probably well back into the 1920s. Overall, the SLD record shows a general decline between the 1920s (20.6 cm) and the beginning of the 2000s (5 cm), after which the trend reverses until 2016 (15 cm). Regardless of the complexity, in what follows, we succinctly catalogue the relations between the trends in SLD, SC flow and species migration into the following three distinguishable time periods: 1923–1980, 1980–2001 and 2001–2016. Particularly, we provide a climatic interpretations for these relationships using well-documented sea level trends and their regional to distinct/global climatic contexts.

### 1923–1980

The SL rise in the northern IO was moderate compared with the higher rates in Port Said from 1923 to 1960, resulting in a sharp decline in SLD values. The high rate of SL rise throughout the MS during the same period was associated with a negative NAO index^24,25^. From 1960 to 1980, stable SL trends at Port Said and Port Suez resulted in a negligible SLD trend (Fig. 3a). Although the reasons for the stable conditions in Port Suez during this period are unknown, those at Port Said were probably associated with the positive NAO index accompanied by atmospheric and hydrographic changes in the MS. These drivers stabilized the SL in the area of Port Said during the 1960s–1990s, despite the rising trend in global SL during this period^21^. From 1923 to 1960, the simulated northward flow transport was ten times higher than the southward flow, with a seasonal duration of 9–11 months. The expansion of the SC during the mid-1950s and moderate SLD values prolonged the seasonal duration of the southward flow by $\sim$ 1 month and tripled the annual average transport, as opposed to the minor rise in the northward seasonal flow (Figs. 2, 3). Regardless of these changes between the two periods (1923–1960 and 1960s–1990s), the high to moderate SLD conditions from 1923 to 1980, accompanied by the smaller dimensions of the SC, contributed to a generally limited mean annual transport in both directions, but with a distinctly dominant northward flow.

### 1980–2000

During the early 1980s, the cross-sectional area of the SC almost tripled (Fig. 1a) and caused a marked increase in northward and southward transports (Figs. 2b and  3b). In general, during the period 1980–2000, the seasonal southward flow regime became more comparable to the northward flow because of the following SL changes on both sides of the SC: The shift in the formation site of eastern MS deep water from the Adriatic to the Aegean Sea, also known as the Eastern Mediterranean Transition (EMT)^26^, was accompanied by hydrographic/dynamic changes that abruptly altered the SL in the eastern MS between the mid-1980s and mid-2000s^21–23,27^. The effect of the EMT was observable as a sharp decrease in SL at Port Said between 1987 and 1993, resulting from preconditioning processes to the EMT, followed by a sharp increase that continued until the early 2000s (Fig. 3a), corresponding with the SL trends reported in the Levantine Basin^22,27^. In contrast, between the 1980s and 2000s, a reduction in SL was observed throughout the IO because of its general cooling^28^, associated with a decline in heat transfer through the Indonesian throughflow^28^, changes to winds^29–31^, decreasing shortwave radiation flux because of the volcanic eruptions of El-Chichon in 1982 and Pinatubo in 1991, and increasing aerosol concentrations over Asia^28^. The SL in Port Suez declined from 1993 to 2000 (Fig. 3a), following the reported trend in the northern IO^29,30^; however, our record for 1980–1993 was only approximated and, thus, could not be fully interpreted (“Methods” section). These opposing SL trends on either side of the SC led to a general reduction in the SLD (but with a notable increase from 1986 to 1993 because of EMT preconditioning), which increased the overall southward flow transport, from 150 m$^3$s$^{-1}$ to 210 m$^3$s$^{-1}$, and extended the seasonal duration of this transport from July–September during the early 1980s to June–October by 2000.

### 2001–2016

Heat uptake in the upper equatorial Pacific and its shift to the southeastern IO partially balances the excess atmospheric heat associated with increasing greenhouse gases, demonstrating the ability of the Pacific to regulate global climate^28,32–34^. Particularly, during 2003–2012, a large portion of the heat taken up by the upper Pacific ocean flowed into the southeastern IO through the strengthened Indonesian throughflow, explaining the slowdown in global surface warming during this period^28,32,33^. Despite this large-scale contribution of heat to the southeastern IO, SL variations northward of 20$^{\circ }$S were mostly related to thermosteric component driven by the internal distribution of heat connected to natural variabilities in regional/distant climatic systems that affect the wind patterns over the IO^29–31,35–38^. Specifically, the intensified SL rise in the northern IO over the past two decades has been associated with wind-driven meridional heat transfer and surface turbulent heat flux^30^ or the weakening of the summer Indian monsoon winds^36,38^, which reduced upwelling in the Arabian Sea and its associated southward heat transfer, increasing the heat content in the northern IO. However, the possible effects of the heating of the southeastern IO on the wind patterns over this area, as demonstrated by the weakening south Asian monsoons^39,40^, have not been thoroughly studied. Additionally, the IO warming north of 20$^{\circ }$S was recently linked to anthropogenic greenhouse gas forcing by increasing downward longwave radiation and heat advection from the southeastern IO to the northern IO via the western boundary current^28^. Either as a response to natural wind variability or anthropogenic warming as discussed above, the increase in SL in the northern IO since the early 2000s has occurred at a higher rate than that in the Levantine Basin, reflecting an increase in the SLD from a minimum value of 5 cm in 2001 to 15 cm in 2016 (Figs. 2, 3). As a result, since 2001, the southward flow in the SC decreased by almost half, and its seasonal duration has shrunk to 3 months, despite the expansion of the SC in both 2010 and 2015.

## Implications for species migration

The introduction and establishment of species from the RS to the MS and vice versa, which is facilitated by the SC, is a multi-stage process, where other factors, rather than the SC dynamics, may play important roles. Yet, traversing the SC is one of the major milestone in this consecutive biological selection process, making the focus on the SC dynamics highly beneficial in providing a better understanding of this complicated migration. Indeed, previous studies assessed that most of the alien species that traverse the SC do so by drifting or swimming^2,4,5,41^, where others assumed that the dynamic conditions of the SC and its variations over the years pose an effective physical barrier influencing the type of migrating species and their migration rates and direction (RS to MS or vice versa)^1,4,5,7–10^.

Our results strengthen the above estimations and justify the focus on the SC dynamics. Particularly, the model results indicate that the current in the main canal (excluding the area of the lakes) can be as high as $0.7\,\text {m}\,\text {s}^{-1}$ (not shown). Such strong currents along the 165 km of the SC probably constitute a profound physical barrier to the movement of organisms upstream while enhancing their dispersal in the downstream direction, therefore reinforces the relationship between the migration characteristics and the SC seasonal dynamics. As such, in addition to increasing transport as a result of the expansion of the SC, variations in the duration of the two seasonal episodes, which are affected by SLDs, and their synchronization with biota life-cycles, such as breeding, spawning, migration periods, could determine the ability of a species to migrate between the two seas. Where, during high-stand SLD conditions, the seasonal window of northward flow is prolonged and synchronized with the shortened duration of the southern flow episode. Therefore, the number of species capable of migrating from the RS to MS would increase. Similarly, a southward species migration is potentially enhanced during low-stand SLD conditions. In that sense, the relatively short duration and negligible transport of the southward flow that existed during high-stand SLD conditions before the 1980s (Figs. 2, 3), likely significantly limited the number of species capable of traversing the SC in that direction, while supporting the theory of a northward unidirectional migration through the SC for this period of time. Moreover, the significant increasing northward transport since the 1980s corresponds well with the apparent migration rate to the eastern MS ever-since. Our results also appear to justify concerns raised by the scientific community regarding the future impacts on biodiversity and the ecological system of the MS of species migration from the RS^13^, given that the inauguration of the New Suez Canal in 2015 increased northward flow transport by $\sim$ 60% (Fig. 2b). However, although long-term trends indicate a clear association between canal expansion and an increase in the rate of species migration through it^42,43^, this relationship is not guaranteed with respect to future trends, as other factors may influence the migration process. (e.g., reduction in pool of potential non-indigenous species). In fact, there are mixed reports regarding the recent migration trends, with some indicating a general slowdown in the rate of species entry into the Mediterranean^6,44^, and others pointing to an increasing introduction rate of fish via the SC^45^.

Another possible contribution to the characteristic migration process is evident from the summertime southward flow regime. Specifically, the flow dynamics in the SC since the early 1980s are likely to have supported enhanced migration rates in both directions rather than unidirectional migration. First, during the early 1980s, the cross-sectional area of the SC almost tripled (Fig. 1a) and caused a marked increase in transport in both directions (Figs. 2b and  3b), not only offering a physical basis for the reported increasing invasion rate to the MS^1^, but also supporting the increasing probability of southward transitions. Second, the deepening of the SC (Fig. 1a), which was previously thought to increase the number of species able to migrate from the RS to the MS^11^, is also likely to have been similarly appropriate for southward migration. Third, between the mid-1980s and mid-2000s the seasonal southward flow regime became more comparable to northward flow, in terms of seasonal flow’s magnitude and duration, due to the low-stand SLD values prevailed during this time (associated with the IO cooling and the EMT, see previous section). These changes in southward flow all have the potential to influence southward migration. If the asymmetric conditions limit southward migration to large pelagic predators that are strong swimmers^9,10^, since the removal of the dynamic barrier that prevailed during the period 1923–1980, any migrating species, regardless of its swimming capabilities, can be sucked into the canal from the MS and freely drift towards the RS.

Since 2001, the decrease in southward flow rate and its shortened duration, which was driven by the intensified SL rise in the northern IO (see previous section), somewhat reduced the potential for migration in a southerly direction. Yet, it should be noted that the southern flow conditions during this period correspond to those recorded during the early 1980s, which still allowed for a southward migration, much more than the 1923–1980 conditions prevails, which were characterized by the unidirectional migration. However, if similar SL trends on both sides of the SC persist, we predict that seasonal transitions to a southward flow will cease by 2040, and the seasonal time window available for MS to RS migration will close. This time duration is short compared with the duration of climate trends that affect SL rise in the IO, especially if the trends are connected to greenhouse gas forcing. Indeed, the CESM-LE projection predicts that, at current emission levels, warming of the southeastern IO will become more frequent throughout the 21st century^28^, potentially resulting in a further increase in SLD values. However, short-term SL variability in the MS might enable southward migration even at high-stand SLD conditions (i.e., as a response to the extreme negative NAO index events that caused the significant SL rise during 2009/2010 and 2010/2011^20,46^).

## Conclusion

We used extensive numerical modelling setup of the SC dynamics and different databases to explore novel interpretations of the migration characteristics through the SC using well-documented sea level trends in their regional and global climatic contexts. Particularly, our results support a potential increase in the rate of southward migration from the MS to the RS through the SC since the 1980s. Aside from the contribution of the expansion of the SC, the potential increase in the species migration to the RS from 1980 to 2001 was also related to cooling of the IO and the EMT, which oppositely affected the SL trends at either end of the SC. The probability of southward migration reduced somewhat after 2001, despite the further expansion of the SC, because of the accelerated SL rise in the northern IO, which was associated with either decadal-multidecadal wind pattern variability over the IO or to a shift in Indo-Pacific heating resulting from anthropogenic greenhouse gas forcing. It is estimated that, if SL trends persist, southern migration will become impossible by 2040.

Our results, indicate a potentially enhanced migration through the SC since the 1980s, which corresponds well with the massive inflow of RS species to the MS, but are not reflected by the reported low-level migration rates to the RS. A review of RS literature back to the 1980s yields only a few known cases of MS species becoming established in the RS (e.g., Mezger et al.^47^ and Malaquias et al.^48^); contrary to previous reports indicating the southward migration before the 1980s, these species lack the necessary swimming capabilities to traverse the SC upstream and, thus, possibly drifted through it. If these cases indicate a possible change in the characteristics of MS to RS migration, more MS species might have invaded the RS in the same way. Another reason for the asymmetric/unidirectional migration reported in the literature, and in opposition to our prediction, is that there are other causes of this asymmetry, other than the SC dynamics, that were not considered here, such as environmental conditions, life cycles and community structure. Namely, although the physical barrier to southward migration has been reduced, MS species still fail to establish successfully in the RS. Yet, the MS species can probably tolerate the temperature range in the upper layer of the northern RS (22–28 °C^49^ compared with 17–30 °C in the MS), whereas the salinity in the northern RS is 40–40.5 psu compared with a maximal salinity of $\sim$ 39.8 psu in the southeastern Levantine Basin. Moreover, the RS-MS hydrographic differences will probably continue to decrease, due to long-term salting and warming trends of the southeastern Levantine Basin^50^. This somewhat excludes RS-MS hydrographic differences as a reason for the asymmetric migration. Another reason could be related to the time lag between the establishment of the species in the RS and their discovery^51^, which, among other factors, is inversely proportional to scientific effort, which in turn is much more intense in the MS than in the RS.

## Methods

In this section we describe our extensive modelling set-up and the reconstructions of the sea levels at the Ports of Suez and Said, the SLD and the flow transport records through the SC.

### Sea level record

The SL records at Ports Said and Suez (Fig. 3a) were computed from in situ SL tidal gauge data taken from the Gulf of Aden (GOA), Alexandria, and Port Said combined with satellite absolute dynamical topography (ADT) data from the GOA and the northern RS, and published sporadic sea level data that were measured at Port Suez. In addition, for better interpretation of the SL results, we utilized the sea level data measured at several locations along the Israeli coastline and historical data taken along the RS. The SL records of Port Said and Port Suez were achieved as follows:

#### Port Suez

Figure 4c shows the computed SL record in Port Suez (also shown in Fig. 3a), including the different sources that contributed to the dataset from which our computational record was constructed. The sea level data from Port Suez were poor, and the last published data were from 1986^52^. Therefore, the SL record at that location was reconstructed based on the in situ SL tidal gauge data taken from the GOA^53,54^ combined with satellite absolute dynamical topography (ADT) data from the GOA and the northern RS (the altimeter products were produced by Ssalto/Duacs and distributed by Aviso, with support from Cnes; http://www.aviso.altimetry.fr/duacs/), and the published sporadic SL data that were measured at Port Suez^52,55–57^.

Although more than 2000 km separate Port Suez from the Port of Aden, satellite SL data (Fig. 4b) and published historical data (Fig. 4a) revealed that the SL boundary conditions at the GOA have a profound impact on the long-term, annual mean, and seasonal SL variabilities in the RS (Supplementary Information). For the reconstruction of the SL record in Port Suez, we used two assumptions (Supplementary Information): (i) the similarity of the long-term SL trends in Port Suez to the observed trends in the GOA, as validated based on satellite data for the years 1992–2016 for the northern and central RS; and (ii) the unique seasonal SL conditions in Port Suez and throughout the RS are a robust characteristic, and therefore, the inter-annual variability of the mean seasonal values can be ignored, and a mean seasonal SL anomaly could be used for the reconstruction of the SL record in Port Suez. This assumption relies on the actual measurements existing for Port Suez (Fig. 4a) and on the existing evidence that this characteristic is driven by more stable regional, rather than local, climatic forcing (i.e., Indian monsoon seasonality).

To reconstruct the long-term monthly mean SL conditions at Port Suez, we first calculated a continuous annual mean SL record between 1879 and 2016. The SL data between the years 1992 and 2016 were completed using the annual mean absolute dynamical topographic satellite data (Fig. 4c). The accuracy of the satellite data was validated and compared with the SL data measured by tidal gauges that were placed at two different locations in the GOA, one in Port Aden (Fig. 4b) and the second in the Port of Djibouti (not shown). For both locations, the annual mean SL differences between the satellite data and the in situ measurements were less than 0.35 cm. There were two extended periods in which data were missing from the SL record of Port Aden: between 1894 and 1916, and between 1970 and 1992. Without any other reliable SL data, these gaps and other short-term data losses in the annual mean SL record from Port Aden were filled using linear interpolation.

In a second step, for a better representation of the seasonal SL pattern in Port Suez, we superimposed the annual mean SL record from the GOA, which was determined in the previous step, with the calculated mean seasonal SL anomaly conditions at Port Suez. The latter was calculated based on 41 years of measurements that were collected in Port Suez during the following periods (1923–1929 and 1931–1946)^56^, (1923–1946 and 1956–1966)^55^, (1924–1937)^57^ and (1980–1986)^52^. The first three data sets cover 34 years of measurements in total (1924–1937 and 1956–1966) and showed similar mean SL anomalies (Fig. 4a); therefore, it was reasonable to take a simple average of the three sources for the representation of these periods. A final mean seasonal SL anomaly for Port Suez was calculated by the following weighted average: $\frac{7}{41}Y_{80-86}+\frac{34}{41}Y_{23-66}$, where $Y_{23-66}$ represents the mean seasonal SL anomaly in the earlier period as described above, and $Y_{80-86}$ is the mean seasonal SL anomaly for the years 1980–1986^52^.

#### Port Said

**Figure 5:**
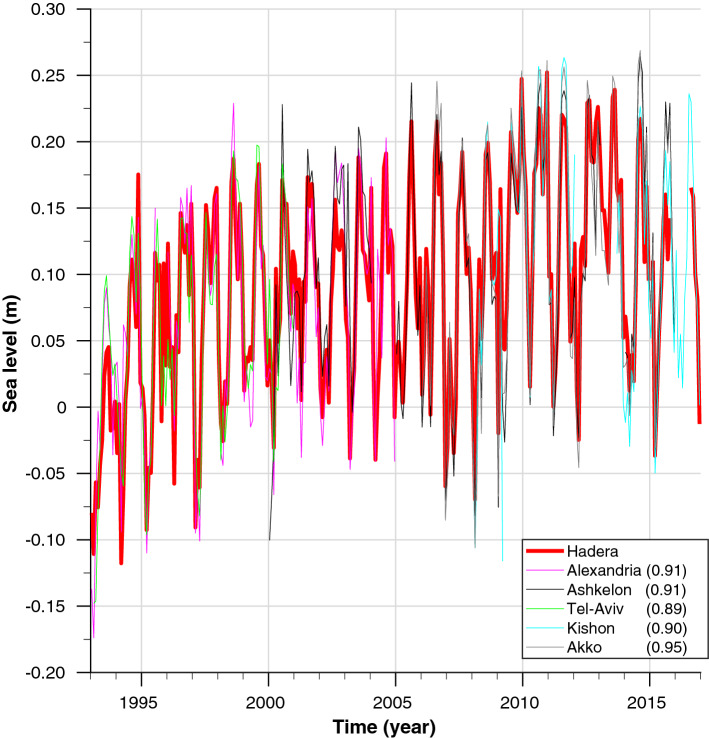
Robustness of SL trends along the eastern Levantine Basin. Monthly mean SL data measured by tidal gauges located at Hadera for 1993–2017 (red) compared with other SL trends along the Israeli Mediterranean coastline (Ashkelon (black), Tel Aviv (green), Kishon Port (cyan), and Akko (gray)) and at Alexandria (magenta). Values in parentheses in the figure legend are the calculated correlation coefficients of the different datasets with respect to the Hadera record. Data from Hadera were taken from the Mediterranean GLOSS $\#$80 station operated by Israel Oceanographic & Limnological Research, Alexandria data are from PSMSL^53,54^, Ashkelon and Akko time series were provided by the Survey of Israel, and data from the Kishon were measured by Israel Oceanographic & Limnological Research and were kindly provided by the ISRAEL PORTS Development & Assets Company Ltd.

SL data from Port Said were only available for 1923–1947; however, there was a long-term SL record from a nearby monitoring station in Alexandria covering the period 1944–2006^53,54^, which as demonstrated below, can be used to complete the sea level record of Port Said. Fig. 5 presents the monthly mean SL data for the years 1992–2016 taken from tidal gauges located in Alexandria and along the Israeli coast. The different records were fit to the SL from the Hadera station (northern Israeli coastline) by only changing their reference level [i.e., by the subtraction/addition of a specific constant value (Hadera was chosen because it has a complete SL record from 1992 to 2016)]. From that figure, it can be seen that the SL records along the eastern Levantine coast share almost identical seasonal (amplitude and phase) and long-term variability. In addition, the annual mean absolute dynamical topography records for 1992–2016 from Alexandria and Port Said show almost constant SL differences of $\sim$ 22.7 cm with a S.D. of 0.7 cm, and a correlation coefficient between the SL time series from the two locations of 0.97. As such, we conclude that the record from Alexandria accurately represents the seasonal and long-term conditions in Port Said. A complete SL record for Port Said between 1923 and 2016 was obtained by interweaving the tidal gauge SL records from Port Said (1923–1947), Alexandria (1948–2006), and the absolute dynamic topography satellite data for 2007–2016 (altimeter products by Ssalto/Duacs and distributed by Aviso, with support from Cnes; http://www.aviso.altimetry.fr/duacs/). Other short-term gaps/missing data in the SL record were filled using linear interpolation.

#### Calculation of SL differences between the RS and MS sides the SC

Our findings show that the SLD values affecting the flow through it. To calculate the SLD record, it was necessary to place the reconstructed SL records from these two locations on the same reference level. The mean SLD for the years 1980–1986 was set to 11.9 cm, as previously calculated^52^, which is the latest record available. However, our SL record showed a similar general decrease in the mean SLD between the 1920s and the 1980s, as previously reported (Fig. 3). More specifically, the calculations of the mean SLD based on our SL record were close to earlier published values, including 12.7 cm for 1955–1963 compared with 12.3 cm estimated by Morcos and Gerges^58^, 20.2 cm for 1924–1937 compared with 22.4 cm given by Morcos^57^, and 20.6 cm for 1923–1925 compared with 24 cm estimated by Lisitzin^59^.

### Numerical simulations

The numerical simulations of the SC were conducted using the Massachusetts Institute of Technology general circulation model (MITgcm)^60,61^. The model domain covers the entire SC beginning at the southern edge of Port Suez to the northern edge of Port Said (Fig. 1b). The horizontal resolution was 20 m across and 100 m along the canal, and the water column was divided into 12 vertical layers, each 2 m thick. The time step was 10 s. The model solves the hydrostatic primitive equations and uses the incompressible fluid, Boussinesq approximation and a linear free-surface condition.

The modelling effort was divided into four series of numerical simulations: R1920–R2015 (or ‘R’ series) simulated the dynamics of the SC during its different construction and development stages and as a response to its various forcing parameters described below; SL1920–SL2015 (or ‘SL’ series), were used to explore the relative importance of the differences in sea level between the two ends of the SC compared with other existing forcing parameters (e.g., salinity and temperature distributions along the SC and wind stress); two matrices of experiments, SLMATRIX and RMATRIX, were conducted to reconstruct a time series of the monthly averaged flow transport through the SC from 1923 to 2016, based on estimated SL records at each end of the SC.

**Figure 6:**
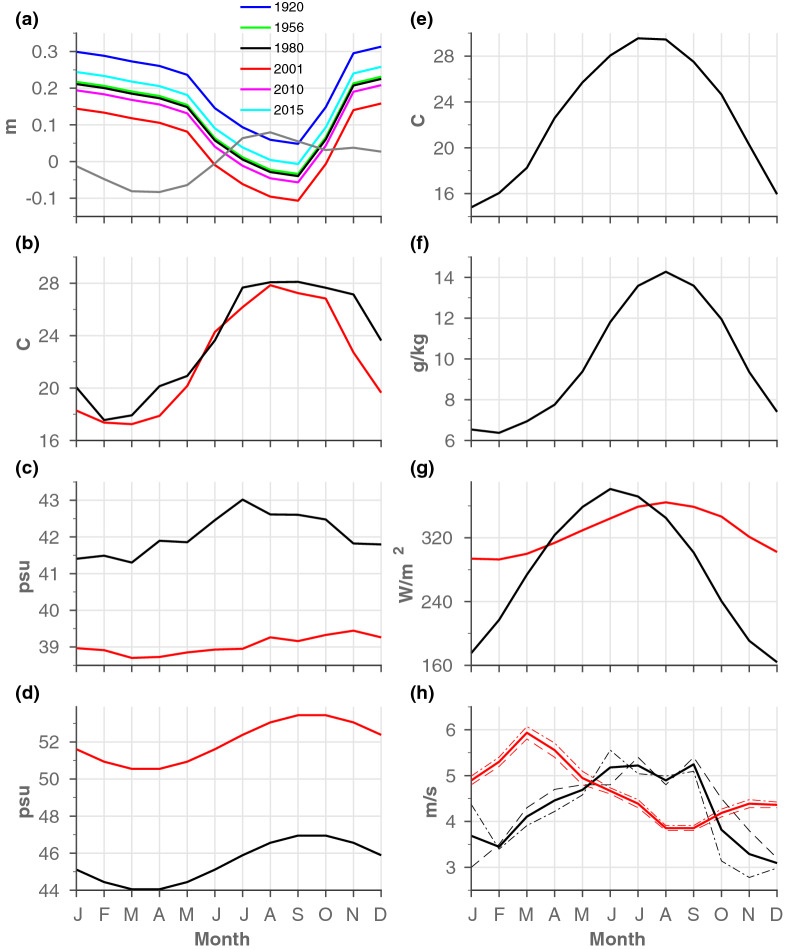
Climatological forcing parameters used in the numerical simulations in this study. (**a**) SL boundary conditions at Port Said (gray) and Port Suez (coloured). For Port Suez data, 6 years are shown that represent the different stages of construction of the SC, to indicate long-term SLD trends. (**b**) Temperature and (**c**) salinity conditions at Ports Said (red) and Suez (black). (**d**) Seasonal salinity values used for the relaxation term in the GBL area for 1923–1955 (red) and 1956–1979 (black). (**e**) Air temperature, (**f**) atmospheric specific humidity, (**g**) downwards longwave (red) and shortwave (black) radiation fluxes and (**h**) meridional wind velocities at Ports Said (red) and Suez (black). The thin dashed^69^ and dashed-dotted^70^ lines represent the measurements taken during different periods, and the thick lines are the averages of the two data sets.

Depending on the specific configuration, all or any combination of the following forcing parameters might apply: at the southern and northern open boundaries of the SC, there are ‘sponge layers’, where relaxations of temperature, salinity, and sea level occur (Fig. 1b). In the Great Bitter Lake (GBL) area (middle SC, Fig. 1b), a relaxation of seasonal salinity values was used to compensate for the possible effects of a salt flux from the bottom salt deposits, which was not explicitly simulated. At the surface, net evaporation, surface heat flux components, and wind stress were calculated in situ by the model based on atmospheric data and using bulk formulas^62,63^. The actual values of the above forcing conditions are presented in Fig. 6 and the different simulations are described in more detail below.

#### Bathymetry

Overall, the dimensions of the canal increased from about 8 m deep, 22 m wide at the bottom and 60–90 m wide at the surface, when it was opened, to today’s dimensions of 24 m deep, 121 m wide at the bottom, and 313 m wide at the surface (Fig. 1a). Until recently, the canal has permitted only single lane traffic with the exception of five short bypasses. In 2015 a new 35 km section was opened in parallel to the canal (Fig. 1b).

A published dataset of the bathymetry of the SC as a whole could not be found, and there is only one historical bathymetric chart of the GBL area^64^. Therefore, a realistic set of bathymetries that accounted for the expansion of the SC over the years was indirectly obtained using information from different sources. First, the shoreline of the SC was digitized from satellite photos taken before and after the last major construction stage in 2015 (final bathymetric results are shown in Fig. 1b). In all cases, the slopes at the sidewalls of the SC were assumed to have a gradient of 0.25, create a uniform trapezoidal cross-sectional shape and appear to have similar dimensions to the slopes given by the Suez Canal Authority (http://www.suezcanal.gov.eg/English/About/SuezCanal/Pages/CanalCharacteristics.aspx, also see Fig. 1a). The GBL bathymetry data are based on a survey conducted in 1925^64^, reaching a maximum depth of 12 m, similar to the maximum depth in the main SC during that time. We assumed that, during the different developmental stages of the SC, the center of the GBL was dredged to the depth of the main canal to allow for maritime transport along the SC. Therefore, in all cases, the bathymetry values at the center of the GBL were set to the maximal depth values existing along the SC.

#### Salinity values at the GBL (used in ‘R’ series and in RMATRIX only)

As mentioned above, in addition to sea level variability at its two ends, which being the focus in this study, other factors may influence the dynamics of the SC (e.g., salinity differences between the two end of the canal), and therefore their relative important should be considered. To realistically simulate the salinity conditions in the GBL (Supplementary Information), a relaxation to the reported salinity values was used to compensate for the possible effects of a salt flux from the bottom salt deposit at this location. In experiments R1920 and R1956, we relaxed the sinusoidal seasonal salinity signal with maximal salinity values from September-October and minimum values from March-April, with seasonal values ranging from 50.5–53.5 psu and 44.5–47 psu, respectively (Fig. 6d). For the experiments R1980–R2015, the salinity in the GBL was relaxed to a constant value of 44.5 psu, which was the latest value reported in the literature and measured during the 1980s^65^. However, it was expected that the decline in the GBL salinity would have continued between 1980 and 2015, caused by either further leaching of the salt bed or expansion of the SC. Therefore, we believe that the 44.5 psu value represents an upper value for the salinity in the GBL from 1980 to 2015. However, the water in the GBL should be saltier than that at the northern tip of the Gulf of Suez, which was $\sim$ 42 psu during winter months and higher than the salinity values of $\sim$ 39 psu that exist in the MS (Fig. 6c), which occur when the flow regime along the SC switches from a northward to southward flow.

#### Hydrographic boundary conditions at Ports Said and Suez (used in ‘R’ series and in RMATRIX only)

At either end of the SC, we applied a sponge layer (Fig. 1b) in which there were relaxations to the climatological monthly average salinity, potential temperature, and sea level conditions. The temperature and salinity boundary conditions are shown in Fig. 6b,c, respectively. At the northern boundary of the SC, the climatological temperature and salinity values for MS water were taken from the SeaDataNet database^66^. Unfortunately, we could not find long-term records of salinity and temperature from the Gulf of Suez region. Therefore, the southern boundary conditions were based on temperature and salinity data that were measured for a relatively short time between April 1999 and June 2000 at the northern tip of the Gulf of Suez^67^.

#### Sea level boundary conditions at Ports Said and Suez (used in ‘R’ and ‘SL’ series)

The sea level boundary conditions are shown in Fig. 6a. The computed seasonal cycles were based on the climatological SL anomalies at Port Said and Port Suez. In each experiment, the northern boundary conditions were set to the climatological SL anomalies at Port Said and, therefore, all experiments had annual mean SL values equal to zero. The seasonal cycle in Port Suez was shifted by different constant values to account for the long-term trends in the SLD (Fig. 6a). These annual mean SLDs were based on the SL records from Port Suez and Port Said and were in close accordance with previous SLD estimations (Fig. 3).

#### Atmospheric conditions (used in ‘R’ series and RMATRIX only)

Evaporation, surface heat flux components, and wind stress (for the prescribed wind vector components) were calculated in situ by the model based on atmospheric data and using the bulk formulas described by Large and Pond^62,63^. The surface monthly average climatological data are shown in Fig. 6e–h. The atmospheric fields comprise sea level air temperature, specific humidity, downward longwave and shortwave radiations, and meridional wind intensity. With the exception of the wind intensity, all the applied atmospheric fields varied in time and were uniform in space. Climatologies for the specific humidity, longwave radiation, and shortwave radiation were based on NCEP data^68^. The climatological air temperature for the SC was determined from measurements at Al Adabiyah, which is located at the northern tip of the Gulf of Suez, and measurements at Ismailia, which is located in the center of the SC. The air temperature climatologies for both locations are available at https://www.yr.no/place/Egypt/Bur_Said/Qaryat_al_Jam. The climatological meridional wind intensities vary linearly along the main axis of the SC between Port Said and Port Suez. Wind data for Port Said and Port Suez were taken as averages of the wind at these two locations given by Sharaf El-Din^69^ and Hatata et al.^70^. Despite the fact that > 50 years separate the two data sets, the wind data from both sources were similar in both intensity and seasonality (Fig. 6h), suggesting that the wind pattern in the area of the SC did not change much over this period of time.

#### Realistic simulations R1920, R1956, R1980, R2001, R2010, and R2015 (‘R’ series)

Simulate the flow in the SC during its different constructive developmental stages and as a response to its various forcing parameters between 1923 and 2016 (results are shown in Fig. 2). These included the gradual expansion of the canal (Fig. 1a), the long-term trends in the annual mean SLD, and the reduction in the salinity in the GBL as a result of the gradual leaching/dissolution of the salt bed and the reduction in the residence time in the GBL because of the expansion of the canal.

#### Sensitivity to sea level differences: experiments SL1920, SL1980, and SL2010 (‘SL’ series)

**Figure 7:**
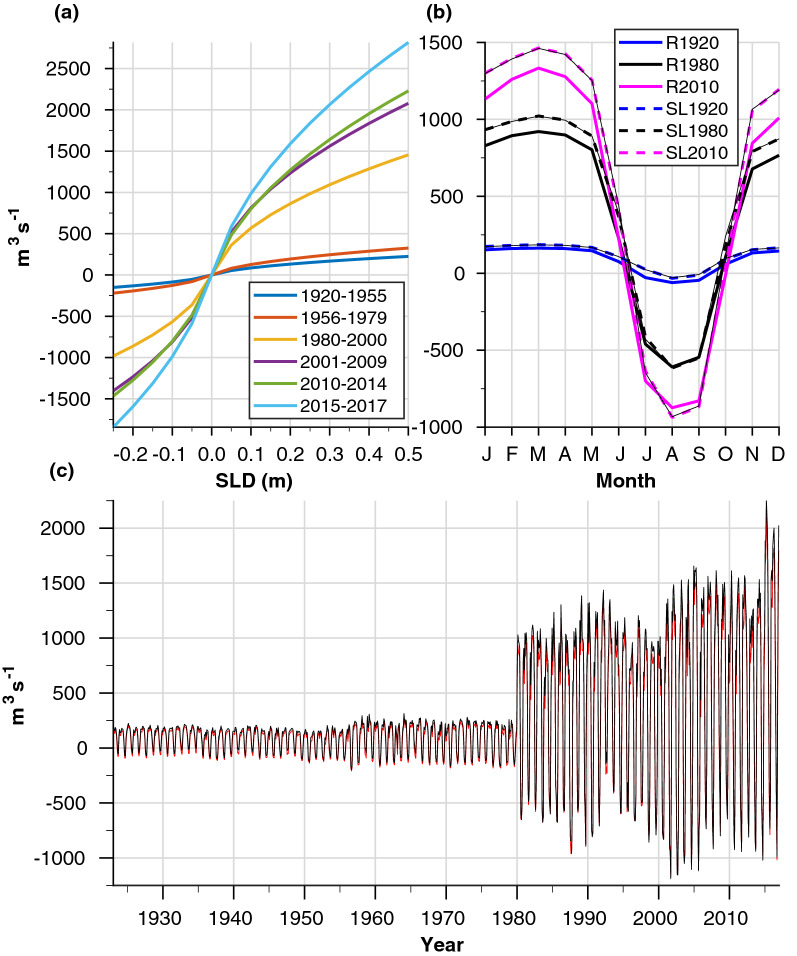
Dominance of SLD conditions on flow transport through the SC. (**a**) Flow transport as a function of the SLD during different periods that follow the developmental stages of the SC (Fig. 1a), as calculate based on the SLMATRIX results. (**b**) Seasonal cycles of the modelled monthly mean transport from experiments SL1920, SL1980, and SL2010 (thick dashed lines) compared with the more realistic results of R1920, R1980, and R2010 (thick solid lines). Thin black lines show reconstructed flow transport calculated based on the SLMATRIX results. (**c**) Reconstructed flow transport records based on RMATRIX (red), as shown in Fig. 3b, and SLMATRIX (black). The SLMATRIX and SL series compare well with the more realistic calculations, but overestimate the northward transport during the winter months.

The bathymetric conditions were realistic as in experiments R1920, R1980, and R2010, but the dynamics were forced solely by the relaxation terms for the SL conditions at the northern and the southern boundaries of the SC, whereas other forcing parameters used in the realistic simulations were not implemented. The fluid is barotropic, where salinity and temperature were constant throughout the entire model domain, with values of 41 psu and 19.5 °C, respectively. The comparison with the ‘R’ series results demonstrated the dominance of the SLD in driving seasonal flow transport through the SC compared with other mechanical and thermohaline/baroclinic forcing parameters (Fig. 7b).

#### Calculations of the transport record—SLMATRIX

This matrix of simulations was used to calculate transport along the SC as a function of its dimensions and SLD (Fig. 7a). The matrix accounted for the results of 96 separated simulations driven by different bathymetry and constant SL boundary conditions. For each bathymetric condition (six overalls, accounting for the different stages of the SC construction between 1923 and 2016), there were 16 simulations driven by different SLDs that varied between − 0.25 m and 0.5 m, in 0.05 m steps. In all cases, transport was calculated after 10 days of simulated results, to represent quasi-steady state conditions. The results show that, for any given bathymetry condition, changes in the transport through the SC because of small SLD perturbations (up to ${\pm }$ 0.05 m in any SL boundary condition) could be safely calculated using linear interpolation.

#### Calculations of transport record—RMATRIX

**Figure 8:**
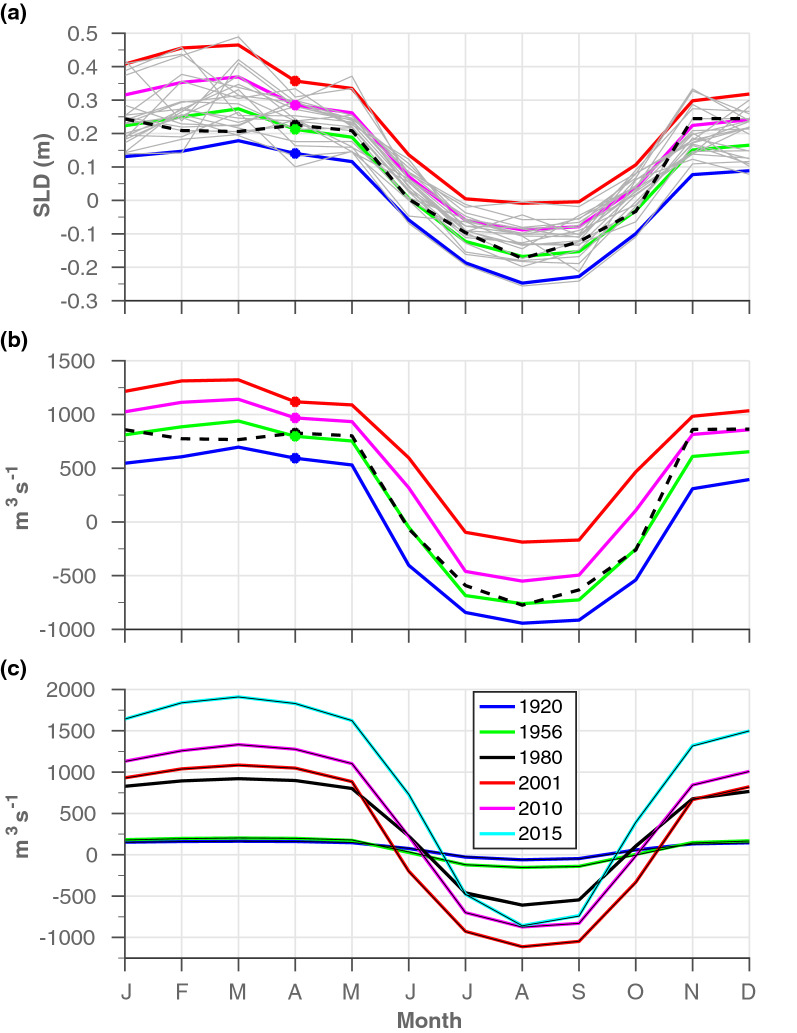
Reconstruction of the monthly mean flow transport during 2000 based on the RMATRIX result: First, the seasonal monthly mean SLD records for 1980–2000 (**a**, thin gray lines) were used to calculate four different SLD boundary conditions for the RMATRIX simulations (**a**, coloured lines). Then, the seasonal monthly mean SLD values from 2000 (**a**, dashed black line) were linearly interpolated, month by month (thick dots in **a** and **b**) using the RMATRIX results (**b**, colored line) to produce the reconstructed flow transport for this specific year (**b**, dashed black line). (**c**) Modelled flow transport results from a realistic series of simulations R1920–R2015 (thick coloured lines) compared with the reconstructed values calculated based on RMATRIX (thin black lines).

This matrix comprised 24 experiments that were used to calculate the flow transport along the SC between 1923 and 2016 (red line in Fig. 7c, also shown in Fig. 3b). The experiments were forced by the same forcing parameters as in experiments R1920–R2015, with the exception of the sea level boundary conditions. For each bathymetric condition used in experiments R1920–R2015, we ran a set of four experiments that fully covered the interannual variability in the seasonal SLD for the years 1923–1955, 1956–1979, 1980–2000, 2001–2009, 2010–2015, and 2015–2016. Fig. 8 demonstrates how the SL boundary conditions for the RMATRIX were calculated and how the resulting flow transports from this matrix were used to calculate the record of the monthly mean time series based on the SLD record as shown in Fig. 3. The seasonal flow transport from experiments R1920-R2015 was independently reconstructed using the RMATRIX results at high accuracy (Fig. 8c), indicating that our method was valid.

## Supplementary Information

Supplementary Information.

## Data Availability

All data presented in this manuscript is available from the corresponding author upon request.
