# Supplementary material for: Possible implications of sea level changes for species migration through the Suez Canal
Source: Sci Rep. 2020 Dec 3;10:21195. doi: 10.1038/s41598-020-78313-2 (PMC7713376; doi:10.1038/s41598-020-78313-2)
Supplement: Supplementary file 1 — Supplementary Information. [file 41598_2020_78313_MOESM1_ESM.pdf]

Supplementary Information for "Possible implications of sea level changes for species migration through the Suez Canal"

Eli Biton[1,*]

[1]Israel Oceanographic and Limnological Research, Department of physical oceanography, Haifa 31080, Israel
[*]email: elib@ocean.org.il

Sea Level at Port Suez and Port Said

Although more than 2000 km separate Port Suez from the Port of Aden, satellite sea level data between 1992 and 2016 (Fig. 4b) and published historical data (Fig. 4a) revealed that the sea level boundary conditions at the Gulf of Aden (northern Indian Ocean) have a profound impact on the long-term, annual mean, and seasonal sea level variabilities in the Red Sea. Specifically, based on the satellite data, the sea level differences between the northern Red Sea and the Gulf of Aden were constant at ∼24.3 cm from 1992 to 2016 (Fig. 4b), with a correlation coefficient between the two records of 0.87. This led us to conclude that the sea level trends in the northern Red Sea, as well as in most places along the Red Sea, could be estimated based on the long-term sea level trends in the Gulf of Aden. Unfortunately, the resolution of satellite data was not high enough to detect the sea level conditions in the relatively narrow Gulf of Suez, so it can only be assumed that the long-term sea level variability at Port Suez is similarly affected by the sea level conditions in the Gulf of Aden as the sea level in the northern Red Sea.

In addition to the long-term sea level trends, the published historical seasonal sea level anomalies from several locations along the Red Sea, including in the Straits of Bab el Mandab (i.e., Perim Island, Fig. 4a) and in the Gulf of Aden, all show similar seasonality with maximal values during winter and minimal values during summer (Fig. 4), which makes this seasonal pattern a robust characteristic of the Red Sea and the Gulf of Aden. This sea level seasonality behaves counter-intuitively to what might be expected from other climatic forces that contribute to the sea level seasonality in the Red Sea, including surface evaporation, atmospheric pressure, wind stress, and steric effects[1]. Patzert[1] noted the similarity between sea level conditions along the Red Sea, the Straits of Bab el Mandab and Aden. He suggested that the steric conditions in the Gulf of Aden were forced by Indian monsoon variability; however, he did not specify a mechanism causing the observed steric conditions. Today, it is known that the prevailing winds during the summer Indian monsoon (June-October) cause an upwelling in the Arabian Sea and the Gulf of Aden, as well as affecting the seasonality of the exchange flow through the Strait of Bab el Mandab[2–7]. Moreover, the winds that prevail during summer at the Gulf of Aden are believed to be the cause of the intrusion of the relatively cold Gulf of Aden intermediate water into the Red Sea. The intrusion of this cold water affects the stratification conditions up to the central Red Sea[7] and, therefore, makes the opposite contribution to the steric conditions in the Red Sea to that of the summertime surface heating. Therefore, the monsoonal seasonality affects the stratification conditions in the Arabian Sea, the Gulf of Aden, and the Red Sea and is probably responsible for the summertime minimum seasonal sea level in these areas. Additionally, the sea level of the Red Sea is likely to be barotropically adjusted to the seasonal sea level fluctuations in the Gulf of Aden, because any sea level difference between the Gulf of Aden and the Red Sea is rapidly equilibrated by the residual flow at the straits. More evidence for the remote influence of the northern Indian Ocean on the sea level of the Red Sea is derived from satellite data, which indicate similar sea level trends since the 1990s in these areas (Fig. 4b).

Salinities in the Great Bitter Lake

The salinity field of the Suez Canal is complex and exhibits large spatial variability. The Suez Canal connects two marginal seas that have distinct water properties, with salinities as high as ∼43.5 psu at the southern boundary of the Suez Canal in the Gulf of Suez[8], compared with salinity values of ∼39 psu at the northern boundary of the Suez Canal in the Mediterranean Sea[9]. Another significant source of salinity in the Suez Canal is the salt bed in the Great Bitter Lake and Little Bitter Lake. The combined volumes and surface areas of these two bitter lakes are larger than those of the rest of the Suez Canal[10]; therefore, this lake system constitutes a dominant source of salinity distribution in the Suez Canal. There are several additional sources of salinity along the Suez Canal, but these have only local effects and, thus, were assumed to make negligible contributions to the salinity balance.

During the opening of the Suez Canal in 1869, the salinity in the Great Bitter Lake was as high as ∼70 psu[11]. Since then, the salinity values have gradually reduced, and the latest published salinity measurements, taken during the 1980s, show values <44.5 psu over most of the Great Bitter Lake[12]. Historical data from before the 1980s indicate that maximum

seasonal salinity values in the Great Bitter Lake appeared from September to October (reviewed in Galil[11]). The latest salinity measurements taken in the Great Bitter Lake during the 1980s showed that this seasonality changed, and that the minimum seasonal salinities used to occur during the summer, possibly indicating a more significant relative contribution of the inflow of less-salty Mediterranean Sea water to the salinity balance of the Great Bitter Lake, as was also found in our study.

References

1. Patzert, W. C. Wind-induced reversal in Red Sea circulation. *Deep. Sea Res.* **21**, 109–121 (1974).
2. Smeed, D. Seasonal variation of the flow in the strait of Bab al Mandab. *Oceanol. Acta* **20**, 773–781 (1997).
3. Smeed, D. Hydraulic control of three-layer exchange flows: application to the Bab al Mandab. *J. Phys. Ocean.* **30**, 2574–2588 (2000).
4. Smeed, D. A. Exchange through the Bab el Mandab. *Deep. Sea Res.* **51**, 455–474 (2004).
5. Siddall, M., Smeed, D., Matthiesen, S. & Rohling, E. Modelling the seasonal cycle of the exchange flow in Bab el Mandab (Red Sea). *Deep. Sea Res.* **49**, 1551–1569 (2002).
6. Gupta, A. K., Anderson, D. M. & Overpeck, J. T. Abrupt changes in the Asian southwest monsoon during the Holocene and their links to the North Atlantic Ocean. *Nature* **42**, 354–357 (2003).
7. Biton, E. *et al.* Sensitivity of red sea circulation to monsoonal variability during the holocene: An integrated data and modeling study. *Paleoceanography* **25**, DOI: 10.1029/2009PA001876 (2010).
8. Gab-Alla, A. A. F. A., Mohamed, S. Z., Mahmoud, M. A. M. & Soliman, B. A. Ecological and biological studies on some economic bivalves in Suez Bay, Gulf of Suez, Red Sea, Egypt. *J. Fish. Aquat. Sci.* **2**, 178–194 (2007).
9. Simoncelli, S., Schaap, D. & Schlitzer, R. Mediterranean sea - temperature and salinity observation collection v2. In *10.12770/8c3bd19b-9687-429c-a232-48b10478581c* (2015).
10. Morcos, S. A. & Soliman, G. F. Salt content and water budget of the Suez Canal. *J. King Abdulaziz Univ. Sci.* **12**, 83–98 (2001).
11. Galil, B. S. *Bridging divides: Maritime canals as invasion corridors*, vol. 83, chap. The marine caravan - the Suez Canal and the Erythrean invasion, 207–300 (Monographiae Biologicae, 2006).
12. Soliman, G. F., Morcos, S. A. & Helali, N. A. The exchange of water between the Mediterranean and the Red Sea through the Suez Canal. *Bull. Inst. Ocean. Fish* **14**, 205–223 (1988).
